# Assessment of subclinical left ventricular systolic dysfunction in patients with type 2 diabetes: Relationship with HbA1c and microvascular complications

**DOI:** 10.1111/1753-0407.13369

**Published:** 2023-02-22

**Authors:** Yanyan Chen, Ying Zhang, Yi Wang, Shengjun Ta, Min Shi, Yingni Zhou, Mengying Li, Jianfang Fu, Li Wang, Xiangyang Liu, Zuowei Lu, Liwen Liu, Zeping Li, Jie Zhou, Xiaomiao Li

**Affiliations:** ^1^ Department of Endocrinology, Xijing Hospital Air Force Medical University Xi'an Shaanxi China; ^2^ Department of Ultrasound, Xijing Hospital Air Force Medical University Xi'an Shaanxi China; ^3^ Nanchang University Queen Mary School Nanchang China

**Keywords:** global longitudinal strain, glycated hemoglobin, microvascular complications, type 2 diabetes mellitus, ventricular function, 2型糖尿病, 糖化血红蛋白, 微血管并发症, 全局纵向应变, 心室功能

## Abstract

**Background:**

We aimed to examine the association between glycated hemoglobin (HbA1c), microvascular complications, and subclinical left ventricular (LV) systolic dysfunction, and to determine the strength of the correlation in asymptomatic patients with type 2 diabetes mellitus (T2DM).

**Methods:**

Global longitudinal strain (GLS) was employed to assess the subclinical LV function of 152 enrolled T2DM patients with preserved LV ejection fraction, with the cutoff for subclinical LV systolic dysfunction predefined as GLS < 18%.

**Results:**

According to univariate analysis, the reduced GLS exhibited association with the clinical features including HbA1c, triglyceride, systolic blood pressure, fasting glucose, heart rate, diabetic retinopathy, and urinary albumin creatinine ratio (UACR) (all *p* < .05). After the factors of gender, age, and related clinical covariables adjusted, multiple logistic regression analysis revealed the HbA1c (odds ratio [OR] 1.66; 95% confidence interval [CI] 1.30–2.13; *p* < .001), UACR (OR 2.48; 95% CI 1.12–5.47; *p* = .025) and triglyceride (OR 1.84; 95% CI 1.12–3.03; *p* = .017) as the independent risk factors for the reduced GLS. Receiver operating characteristic curve showed a predictive value of the HbA1c for the subclinical LV systolic dysfunction (area under curve: 0.74; *p* < .001).

**Conclusions:**

In asymptomatic T2DM patients, subclinical LV systolic dysfunction was associated with HbA1c, diabetic complications, and triglyceride. More prominently, HbA1c may exert a prognostic significance for the progression of myocardial damage.

## Graphical abstract

Highlights

In asymptomatic type 2 diabetic patients with preserved left ventricular ejection fraction, a negative correlation of glycated hemoglobin with subclinical left ventricular systolic dysfunction was revealed. Furthermore, glycated hemoglobin may make prognostic significance for the progression of myocardial damage.

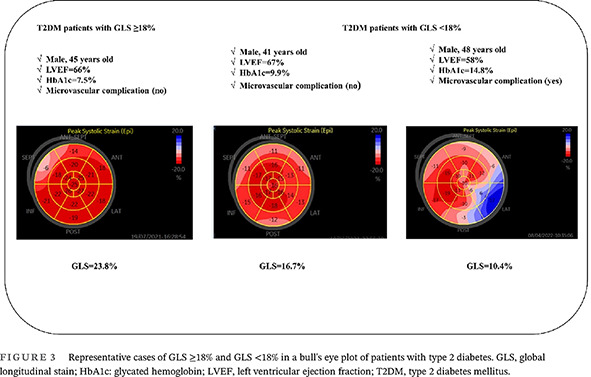

## 摘要

**背景:** 本研究旨在探讨糖化血红蛋白(HbA_1c_)、微血管并发症和亚临床左心室(LV)收缩功能障碍之间的关系, 并确定在无症状的2型糖尿病(T2DM)患者中相关性的强度。

**方法:** 采用全局纵向应变(GLS)评估152例左室射血分数保留的T2DM患者的亚临床左室功能, 亚临床左室收缩功能障碍的临界值预先定义为GLS < 18%。

**结果:** 单因素分析显示, GLS降低与糖化血红蛋白、三酰甘油、收缩压、空腹血糖、心率、糖尿病视网膜病变、尿白蛋白肌酐比值(UACR)等临床特征相关(所有P < 0.05)。将性别、年龄和相关临床协变量因素校正后, 多元逻辑回归分析显示HbA_1c_(OR 1.66, 95%CI1.30~2.10, P < 0.001)、UACR(OR 2.48, 95%CI1.12~5.47, P = 0.025)和三酰甘油(OR 1.84, 95%CI1.12~3.03, P = 0.017)是GLS降低的独立危险因素。受试者工作特征曲线(ROC)显示糖化血红蛋白对亚临床左室收缩功能障碍具有预测价值(曲线下面积: 0.74, P<为0.001)。

**结论:** 在无症状T2DM患者中, 亚临床左室收缩功能障碍与糖化血红蛋白、糖尿病并发症和三酰甘油相关。更显著的是, 糖化血红蛋白可能对心肌损伤的进展有预后意义。

## 1 INTRODUCTION

Diabetes mellitus and heart failure as the mutual risk factors exert an influence on each other,^1^ in which disturbances of cardiac lipid and glucose metabolism are considered the early deterioration events of cardiac function in diabetes mellitus.^2^ The chronic hyperglycemia resulting from insulin resistance or insulin deficiency has been demonstrated to be the starting point of the cascade that launches the diabetes‐related cardiomyopathy.^3^ A linear relationship between glycemic levels and the long‐term mortality of heart failure emerges even before the occurrence of clinical symptoms of diabetes.^4^ The clinical pathologic entity was first proposed for “diabetic cardiomyopathy” as early as 1972.^5^ Most studies prefer support the conventional hypothesis that left ventricle (LV) diastolic dysfunction is the first transformation in cardiac function during the progression of diabetic cardiomyopathy.^6^, ^7^ However, recently, it has been proposed that systole impairment may occur at a more previous stage of the diabetic cardiomyopathy process prior to the detectable transformation of ejection fraction (EF), and advised subclinical systolic dysfunction might as the first indicator of diabetic cardiomyopathy.^8^, ^9^ Under this context, a series of novel diagnostic techniques have been proposed as the deepened research on the evaluation of myocardial function. For instance, the reduced global longitudinal strain (GLS) has been demonstrated to effectively assess global LV systolic function^10^ and is considered a sensitive indicator in identifying subtle variations in LV myocardium. The concept of “common soil” theory indicates a great contribution of the diabetes‐related microvascular malfunction to the adverse myocardium alterations.^11^

On the other hand, multiple studies have confirmed the relation of glycated hemoglobin (HbA1c) to incident cardiac events.^12^ In the UK Prospective Diabetes Study clinical study, each 1% reduction in HbA1c was correlated with a 16% decrease in the risk of heart failure.^13^ Furthermore, in a large cohort study HbA1c >10.0% was correlated with a 1.56‐fold elevated risk of cardiac failure in comparison to HbA1c <7.0%.^14^ However, several investigations have recently stated that the intended glycemic control could not achieve a reduced risk of heart failure and cardiovascular events among individuals with diabetes.^15^ Whether the level of HbA1c and microvascular complications contribute to subclinical impairment of LV systolic function remains to be investigated. Herein, the current study was carried out to investigate the association between HbA1c, microvascular complications and GLS, so as to determine the strength of the correlation in asymptomatic patients with type 2 diabetes mellitus (T2DM) to estimate the independent effect of those on subclinical LV systolic function.

## 2 METHODS

### 2.1 Study population

It was initially designed as a 1‐year cross‐sectional survey, although terminated early because of the outbreak of the COVID‐19 pandemic, only half a year's data results were displayed. Patients defined as type 2 diabetes at the Department of Endocrinology of Xijing Hospital of Air Force Medical University from June 2021 to December 2021 were enrolled. The diagnosis of T2DM was based on the World Health Organization criteria.^16^ Patients with (1) type 1 diabetes; (2) coronary artery disease or other heart disease history; (3) anemia; (4) severe valvular disorders; (5) LVEF <50%; (6) atrial fibrillation; and (7) blood pressure >180/100 mm Hg were excluded. All patients received conventional echocardiography, tissue doppler echocardiography, two‐dimensional speckle tracking echocardiography (2D STE) and bilateral carotid ultrasound examination. The poor echocardiography images were excluded from analysis. Ultimately, 152 subjects were identified and signed informed consent. This study was approved by the local ethics committee of our institution (No. XJLL‐KY20222107).

### 2.2 Patient information

Demographic information covering diabetes duration, gender, age, medication, history of hypertension, and systolic and diastolic pressures was obtained from the electronic medical record system. The fasting blood samples were taken the next morning using an automatic biochemical analyzer (Centrifugation was performed at 4000 g for 5 minutes) and employed to detect traditional lipid profiles, apolipoproteins A1 and apolipoproteins B, and uric acid. Fasting plasma glucose was measured by the glucose oxidase method. The enzyme was used to measure the triglyceride and total cholesterol. Low‐density lipoprotein‐cholesterol (LDL‐C), high‐density lipoprotein‐cholesterol (HDL‐C), and other biochemical indicators were determined by direct analysis method (Hitachi Automatic Biochemical Analyzer, 7170). HbA1c was checked by high‐performance liquid chromatography (Tocho Liquid Chromatograph, G8‐90SL). Height and weight were collected to calculate the body mass index (BMI) by weight/height^2^ (kg/m^2^). Urinary albumin‐to‐creatinine ratio (UACR) was examined using random urine and detected by immunoturbidimetry (COBAS INTEGRA 400 plus autoanalyzer, Germany). The clinical diagnosis of diabetic nephropathy was carried out according to an increase in UACR and, or decrease in estimated glomerular filtration rate (<60 mL/min/1.73 m^2^) with other chronic kidney diseases excluded by an experienced clinician. UACR <30 mg/g in two of three consecutive measurements was defined as normoalbuminuric, and UACR ≥30 mg/g as increased urinary albumin excretion. The diagnosis of diabetic retinopathy was assessed using a fundoscopy performed by an ophthalmologist, with the bias controlled by the double‐blind method. Also, the carotid intima‐media thickness (cIMT) was recorded simultaneously using the high‐resolution B‐mode ultrasonography to obtain the left and right common carotid artery; the maximal intima‐media thickness on both sides of these was recorded and averaged.

### 2.3 Conventional echocardiography

The measurements of all subjects were collected based on the American Society of Echocardiography guidelines,^17^ and every individual underwent transthoracic echocardiography (Philips Healthcare, iE33 system, X5‐1 probe) synchronously connected to the electrocardiogram. LV fractional shortening, LVEF, heart rate, and stroke volume were subsequently analyzed. In addition, peak velocity in early diastole (E‐wave) and late diastole (A‐wave) were measured to calculate the E/A ratio. The early diastolic mitral annulus velocity (E') was measured by the pulsed wave tissue doppler imaging, to obtain the E/E′ ratio that was assessed as an index of LV filling pressures.

### 2.4 Speck‐tracking echocardiography

The 2D STE analysis was performed on each subject, with the 2D gray scale dynamic diagrams of three consecutive cardiac cycles of LV apical four‐chamber view and apical two‐chamber view and apical three‐chamber view collected under calm breathing. The endocardial and epicardial borders were automatically traced using the software of QLAB 8.1 2D strain analysis, to display the 2D strain‐time curve and bull's‐eye plot of 17 segments of LV. The image would be reread if two or more components of the global average strain were missed until no more than one fragment was rejected. The untraceable images of spots resulting from atrial fibrillation were excluded from the analysis. The average value of the three peak strains in systole was employed to evaluate the LV systolic function by calculating the LV GLS. According to the current guidelines of the European Association of Cardiovascular Imaging, subclinical LV systolic dysfunction was defined as GLS < 18%.^17^, ^18^

### 2.5 Statistical analysis

The normal distribution was determined according to the P–P plot and Kolmogorov–Smirnov Test, and a nonparametric test was employed if the assumption of normality was not met. According to the distribution, categorical variables were displayed as percentages n (%) and continuous variables as means ± SD, or median and interquartile range. The comparison of quantitative variables was conducted using Student's *t* or Mann–Whitney U tests. The relationship between continuous variables was assessed by the Pearson correlation coefficients. Categorical variables were compared using Fisher's exact or the Pearson's hi‐square test. The independent risk factors of injured GLS were determined by multivariable logistic regression analysis. The age, gender, and the significant variables with *p* values <.05 in the univariate logistic regression were adjusted by backward stepwise selection, with the odds ratio (OR) and 95% confidence interval (CI) provided. No multicollinearity was detected between variables by variance inflation factor check. The predictive capability of HbA1c was estimated using receiver‐operating characteristic curves (ROC), with the optimum cutoff value, sensitivity, and specificity given. Based on the distribution, UACR was converted into logarithmic form for each analysis. Patients with retinopathy and, or nephropathy were classified as the complications group. To prevent the interobserver and intraobserver variability of GLS, the measurement of GLS was performed by one professional physician. To avoid the ambiguity of negative size to a value, the GLS was displayed in the absolute value form. All statistical analysis was conducted by SPSS statistics version 26.0, with *p* value on two‐sided <.05 regarded as significant.

## 3 RESULTS

### 3.1 Basic features of patients with GLS <18% and ≥18%

According to GLS levels, the basic T2DM patient characteristics were summarized in Table 1. A total of 152 subjects were included, among whom 46.7% suffered GLS <18% (n = 71), with an increased possibility to suffer higher levels of HbA1c and poor blood glucose control, hypertriglyceridemia, and higher systolic pressure and heart rate. Moreover, they showed a higher prevalence of diabetic microvascular complications (neuropathy and retinopathy). No obvious difference was found in gender, age, diabetes duration, BMI, and prevalence of hypertension between patients with GLS <18% and GLS ≥18%, accompanied with the similar levels of conventional echocardiographic parameters and cIMT (all *p* > .05).

**TABLE 1:** Baseline characteristics of T2DM patients with GLS ≥18% and <18%.

Characteristic	Patients with GLS ≥18% (n = 81)	Patients with GLS <18% (n = 71)	*p* value
Male gender, n (%)	52 (64)	45 (63)	.92
Age, years	54.9 ± 13.6	51.1 ± 14.5	.11
BMI, kg/m^2^	23.5 ± 3.6	24. 6 ± 3.9	.09
Diabetes duration, years	9.9 ± 7.2	10.3 ± 7.3	.80
Heart rate, bpm	74.1 ± 11.5	79.2 ± 13.3	**.012**
SBP, mm Hg	130.2 ± 14.1	136.6 ± 21.2	**.028**
DBP, mm Hg	77.1 ± 9.1	79.7 ± 12.7	.15
cIMT, mm	0.41 ± 0.5	0.45 ± 0.3	.64
HbA1c, %	8.0 ± 1.5	9.8 ± 2.3	**<.001**
FPG, mmol/L	10.8 ± 4.6	12.7 ± 5.1	**.015**
Total cholesterol, mmol/L	4.0 ± 1.0	4.1 ± 1.5	.39
HDL, mmol/L	1.2 ± 0.5	1.0 ± 0.3	.063
LDL, mmol/L	2.4 ± 1.2	2.4 ± 1.1	.90
Triglyceride, mmol/L	1.4 ± 0.8	2.0 ± 1.7	**.01**
Apolipoproteins A1, g/L	1.2 ± 0.2	1.1 ± 0.2	.23
Apolipoproteins B, g/L	0.7 ± 0.4	0.7 ± 0.3	.92
Albuminuria, mg/L	10.7 (8.3–14.3)	14.5 (8.4–48.3)	**.01**
UACR, mg/mmoL	1.2 (0.8–2.4)	2.2 (1.4–5.9)	**<.001**
Uric acid, umol/L	320.6 ± 74.1	321.9 ± 90.0	.92
Diabetic nephropathy, n (%)	14 (17)	21 (30)	.072
Diabetic retinopathy, n (%)	6 (7)	15 (21)	**.018**
Hypertension, n (%)	32 (40)	37 (52)	.119
Medical treatment			
ACEI/ARB, n (%)	14 (17)	20 (28)	.108
CCB, n (%)	13 (16)	18 (25)	.156
Statin, n (%)	15 (19)	20 (28)	.159
Insulin, n (%)	40 (49)	43 (61)	.167
DPP‐4I, n (%)	10 (12)	7 (10)	.627
SGLT‐2I, n (%)	9 (11)	5 (7)	.387
GLP‐1RA, n (%)	6 (7)	9 (13)	.277
Metformin, n (%)	60 (74)	45 (63)	.155
**α**GI, n (%)	28 (35)	26 (37)	.792
Echocardiographic indexes			
LV EF, %	60.2 ± 4.3	59.9 ± 5.1	.65
LV FS, %	32.0 ± 3.4	32.0 ± 4.3	.98
Stroke volume, mL	46.2 ± 8.0	48.6 ± 9.8	.12
E/A ratio	1.1 ± 1.9	0.9 ± 0.4	.48
E/E' ratio	9.9 ± 3.8	10.2 ± 3.1	.67
GLS, %	20.5 ± 2.1	15.1 ± 2.2	**<.001**

*Note*: Values are presented as mean ± SD, median (interquartile range), or n (%). Bold indicated value of *p* < .05.

Abbreviations: ACEI, angiotensin‐converting enzyme inhibitor; ARB, angiotensin II receptor blocker; BMI, body mass index; CCB, calcium channel blocker; cIMT, carotid intima‐media thickness; DBP, diastolic blood pressure; DPP‐4I, dipeptidyl peptidase‐4 inhibitor; E/A, peak early diastolic (E‐wave) and late diastolic (A‐wave) velocities ratio; E/E', mitral inflow E and mitral E' annular velocities ratio; FPG, fasting plasma glucose; GLP‐1RA, glucagon like peptide‐1receptor agonist; GLS, global longitudinal strain; HbA1c, glycated hemoglobin; HDL, high‐density lipoprotein cholesterol; LDL, low‐density lipoprotein cholesterol; LVEF, left ventricular ejection fraction; LVFS, LV fractional shortening; SBP, systolic blood pressure; SGLT‐2I, sodium glucose cotransporter 2 inhibitor; T2DM, type 2 diabetes mellitus; TC, total cholesterol; UACR, urinary albumin‐to‐creatinine ratio; α‐GI, α‐glucosidase inhibitor.

### 3.2 Association of clinical features with reduced GLS

**FIGURE 1:**
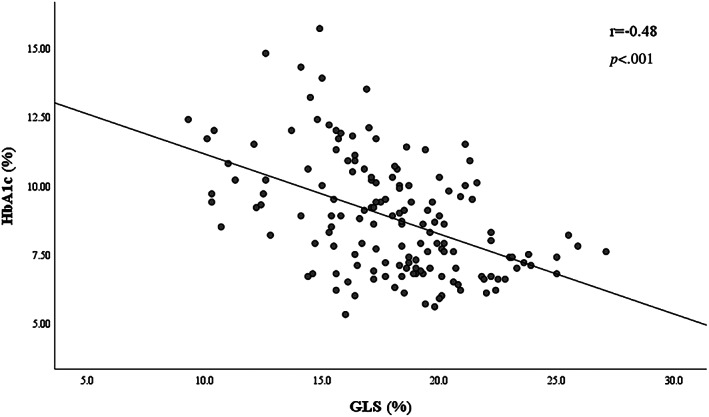
Association between GLS and HbA1c. Scatter diagram showing a significant negative correlation between GLS and HbA1c (*r* represents Pearson correlation coefficients). GLS, global longitudinal stain; HbA1c, glycated hemoglobin.

The comprehensive evaluation of relationship between clinical and biochemical features and GLS was provided in Table 2. Univariate logistic regression analysis indicated the reduced GLS <18% was prominently associated with HbA1c, triglyceride, UACR, systolic blood pressure, fasting plasma glucose, heart rate, and diabetic retinopathy (all *p* < .05). Accordingly, after adjusting for gender, age, and relevant clinical covariables (defined as *p* < .05 in the univariate analysis), the most diabetes‐related clinical features difference was attenuated, whereas the independent association of higher HbA1c (OR: 1.66; 95% CI = 1.30–2.13; *p* < .001), UACR (log‐transformed; OR 2.48; 95% CI: 1.12–5.47; *p* = .025), and triglyceride (OR = 1.84; 95% CI: 1.12–3.03; *p* = .017) with GLS < 18% remained (Table 2). Furthermore, Pearson correlation analysis revealed a negative correlation of the HbA1c level (*r* = −0.48, *p* < .001) with GLS on plotting in the scatter diagram (Figure 1).

**TABLE 2:** Logistic regression analysis to identify GLS <18%.

Variables	Univariate			Multivariate		
	OR	95% CI	*p* value	OR	95% CI	*p* value
Gender (female)	1.04	0.53–2.01	.92	—	—	—
Age	0.98	0.96–1.00	.11	—	—	—
BMI	1.08	0.99–1.18	.09	—	—	—
Diabetes duration	1.01	0.96–1.05	.80	—	—	—
HbA1c	1.66	1.36–2.04	**<.001**	1.66	1.30–2.13	**<.001**
SBP	1.02	1.00–1.04	**.03**	—	—	—
FPG	1.09	1.01–1.17	**.02**	—	—	—
Triglyceride	1.52	1.10–2.12	**.01**	1.84	1.12–3.03	**.017**
Heart rate	1.04	1.01–1.06	**.01**			
UACR^a^	2.59	1.38–4.89	**.003**	2.48	1.12–5.47	**.025**
Statins use	1.73	0.81–3.70	.16	—	—	—
Insulin use	1.57	0.83–3.00	.17	—	—	—
Metformin use	0.61	0.30–1.21	.16	—	—	—
Microvascular complications (yes)	2.31	1.13–4.74	**.02**	—	—	—
Diabetic retinopathy (yes)	3.35	1.22–9.18	**.02**	—	—	—

Abbreviations: BMI, body mass index; CI, confidential interval; FPG, fasting plasma glucose; GLS, global longitudinal strain; HbA1c, glycated hemoglobin; OR, odds ratio; SBP, systolic blood pressure; UACR, urinary albumin‐to‐creatinine ratio.

^a^ Log transformed; Microvascular complications (diabetic nephropathy and/or diabetic retinopathy).

Bold indicated value of *p* < .05.

**FIGURE 2:**
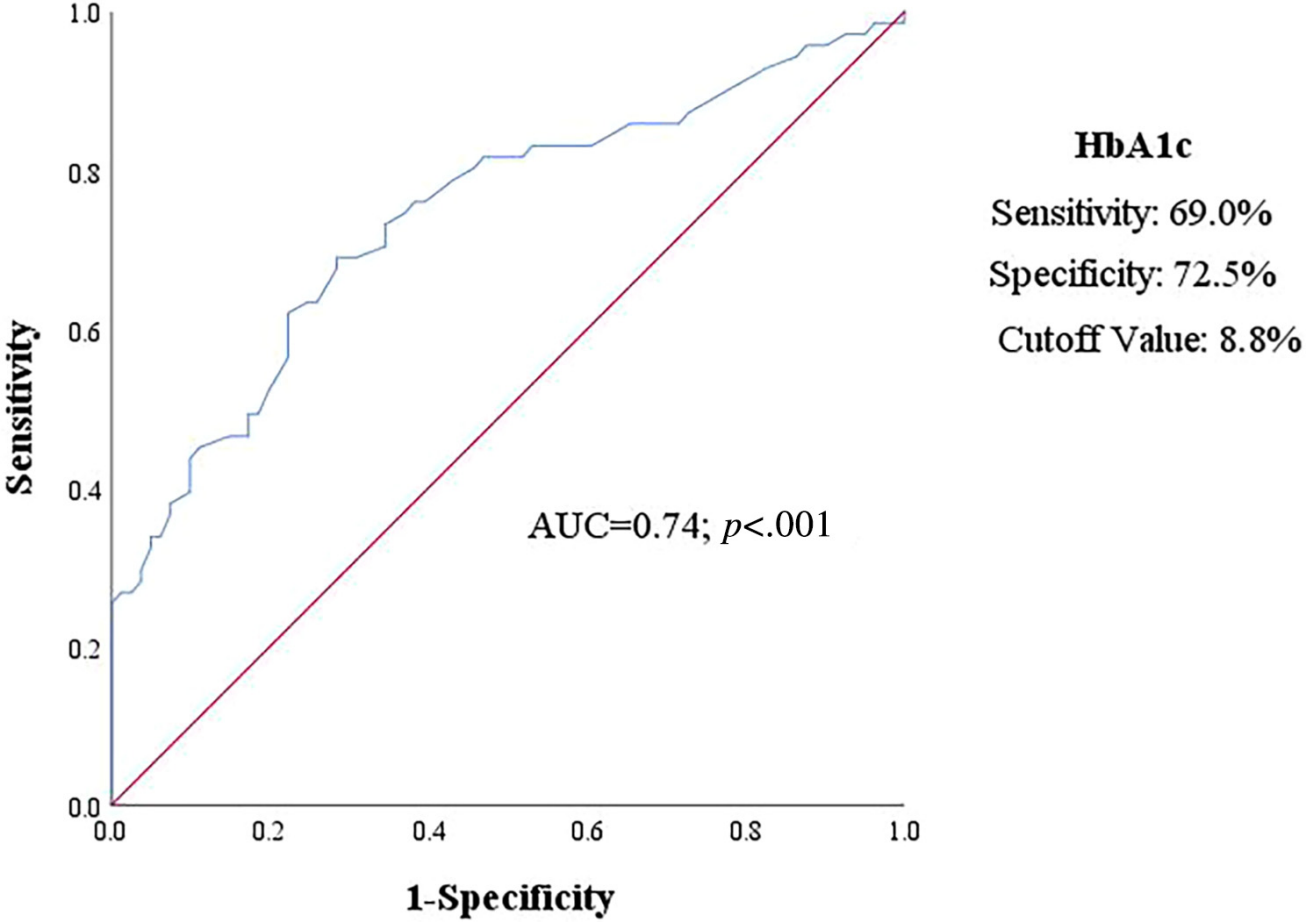
Receiver‐operating characteristic curves for prediction of left ventricular systolic dysfunction in patients with type 2 diabetes using HbA1c. AUC, area under curve; HbA1c, glycated hemoglobin.

The diagnostic performance of HbA1c and the proposed cutoff value was presented in Figure 2 by ROC curves. Notably, the cutoff value of HbA1c (area under the curve = 0.74; *p* < .001) was 8.8%, revealing a sensitivity of 69.0% and specificity of 72.5% for predictive subclinical LV systolic dysfunction.

**FIGURE 3:**
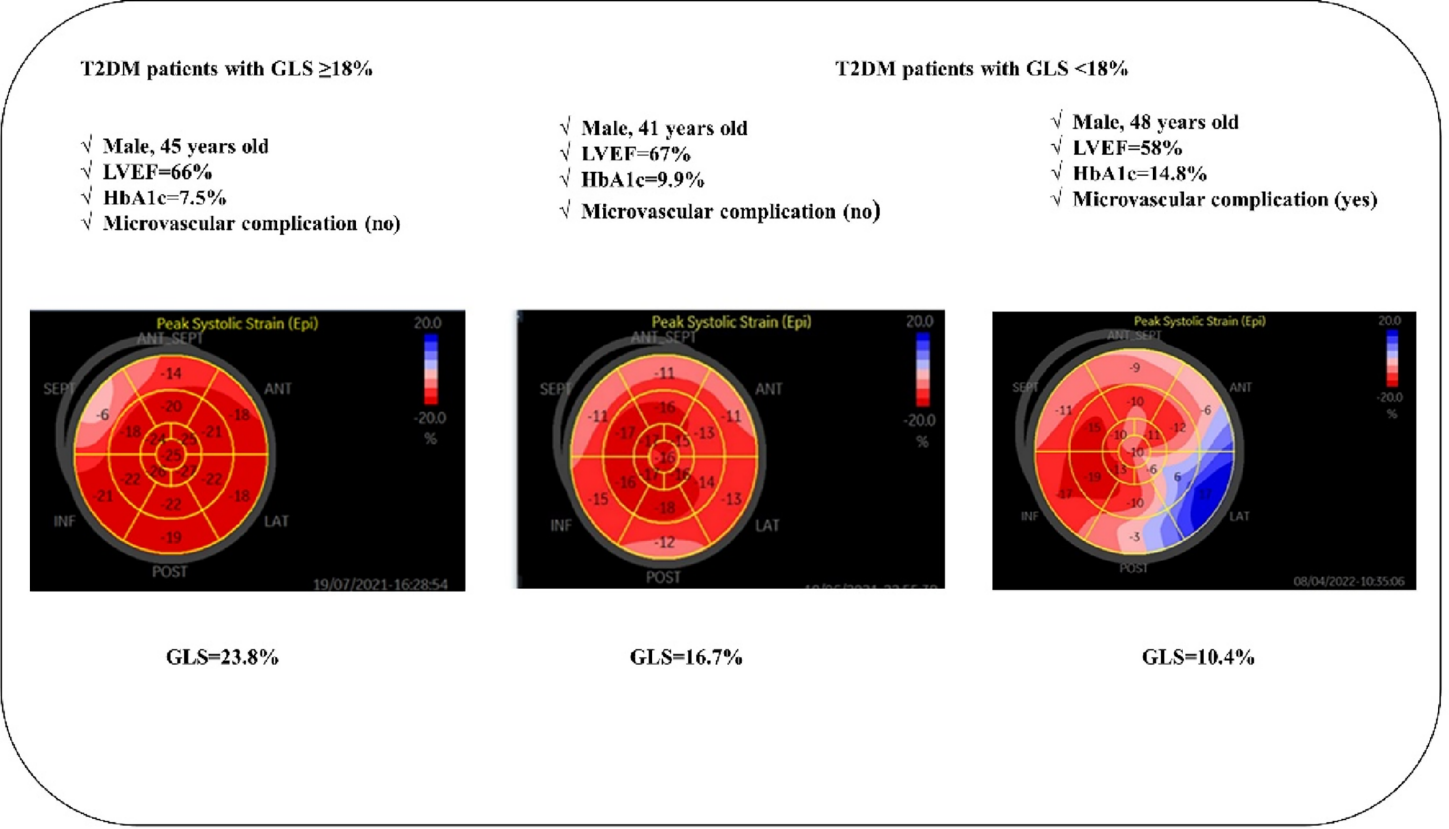
Representative cases of GLS ≥18% and GLS <18% in a bull's eye plot of patients with type 2 diabetes. GLS, global longitudinal stain; HbA1c, glycated hemoglobin; LVEF, left ventricular ejection fraction; T2DM, type 2 diabetes mellitus.

Figure 3 provided the representative cases of GLS ≥18% and GLS < 18% in a bull's‐eye plot of patients with type 2 diabetes.

## 4 DISCUSSION

Our study demonstrated the main potential contributors of suboptimal glycemic control, hyperlipidemia, and microangiopathy to the impaired myocardial systolic function in asymptomatic T2DM patients. More significantly, HbA1c may exert a certain prognostic significance for the progression of myocardial damage.

### 4.1 Clinical risk factors and LV longitudinal myocardial function

In this study, a total of 46.7% of patients exhibited the decreased GLS, which reached an accordance with the prior reports of subclinical LV longitudinal systolic dysfunction in people with diabetes. In various studies, GLS was adopted as the preferential indicator to evaluate the global systolic function, for the longitudinal subendocardium fibers as the most vulnerable part are the first to suffer damage from metabolism disorder in the early stage of the clinical pathologic entity proposed for “diabetic cardiomyopathy.”^19^ The more specific mechanism of this disorder remains unclear. However, metabolic characterizations indicate that chronic hyperglycemia and glucotoxicity exert direct damage on myocardial cells through the accumulated advanced glycosylation end products or lead to myocardial ischemia by eliciting the steady deterioration of endothelial function.^20^ Lipotoxic injury from lipid oversupply is also considered to play a critical role in developing myocardial injury.^21^ Conclusively, glucotoxicity, lipotoxicity, and coronary microcirculation dysfunction might jointly affect longitudinal cardiac dysfunction, manifested by the more advanced transformation in the early form of myocardial deformation.

In addition, research based on the Framingham Heart Study has revealed the significant conjoint associations of hypertension and diabetes with GLS, suggesting a synergistic effects on the reduced GLS.^22^ Ballo et al demonstrated that diabetes, but not hypertension, exerted a negative effect on LV systolic function.^23^ The current study revealed that T2DM patients with reduced GLS preferentially suffered higher systolic blood pressure, compared with those with GLS ≥18%, further validating the adverse influence of hypertension on the subclinical myocardial injury. Moreover, the relationship between carotid atherosclerosis and GLS was also a focus. In a study covering 338 young individuals with concomitant obesity and type 2 diabetes, the increased cIMT was revealed to be independently associated with the reduced GLS,^24^ which, however, was not observed in the present study. This may be related to the fact that thickened cIMT is generally taken as a risk marker for diabetic macrovascular disease,^25^ whereas microangiopathy tends to exert a more prominent effect on the impaired global strain in young subjects. Moreover, another clinical trial by Yamauchi et al demonstrated a close association of heart rate with LV longitudinal myocardial function and highlighted a great contribution of high heart rate ≥70 bpm to early LV damage in asymptomatic T2DM patients.^9^ The consistent result was also revealed by the univariable analysis of this study but weakened in multiple regression analysis, which might result from the higher proportion of patients with GLS ≤18% taking cardiovascular protective medications.

### 4.2 Hyperglycemia and LV longitudinal myocardial function

In this study, it is notable that fasting plasma glucose was associated with reduced GLS, but this did not emerge in multivariate analysis. The underlying pathological mechanism could be unstable fasting glycemia that is susceptible to interference by other stress factors such as diet and medication. Moreover, other investigators have proposed that the cardiomyopathy induced by glucose toxicity could be reasonably considered the cause of cardiac dysfunction in diabetic patients.^26^ For instance, epidemiological studies have demonstrated the association of each 1% increase in HbA1c with the relative risk of cardiovascular disease increase by 18% and with an 8% increase in heart failure among T2DM patients.^27^ The present study found a significant correlation of HbA1c with the reduced GLS, independent of conventional cardiovascular risk factors, which corresponded to the previous research,^28^ demonstrating a close association of poor glycemic control with LV longitudinal dysfunction. And, most important, the results of ROC showed that HbA1c > 8.8% could identify patients with high risk of early alterations of myocardial function. This finding is clinically relevant and supports that HbA1c serves as an independent warning and a screening predictor of early diagnosis of subclinical ventricular dysfunction.

### 4.3 Microvascular complications and LV longitudinal myocardial function

Although the relationship between retinopathy and the heart has been established, the correlation with subclinical LV systolic function remains to be discovered. A cross‐sectional study including 82 T2DM patients demonstrated that GLS was free from the impacts of retinopathy.^29^ Similarly, Pararajasingam et al also pointed out that none of the subgroups of retinopathies had a relation to GLS.^30^ However, increasing studies demonstrated the prominent relation of the diabetic retinopathy to the impaired LV systolic function as evaluated by GLS <18%.^31^, ^32^ Accordingly, out of 71 patients with reduced GLS in present study, only 15 (21.1%) retinopathy patients exhibited GLS <18%. Nonetheless, there remained a significant difference in GLS between patients with or without retinopathy, which further supported the latter conclusion.

More recent evidence suggests that diabetic nephropathy, especially albuminuria, was not only a robust predictor of cardiovascular events in diabetes but more an early marker of widespread vascular injury with high sensitivity.^33^ A study reported the close relation of the declined myocardial flow reserve to albuminuria based on positron emission tomography/computed tomography outcomes.^34^ Additionally, according to the Adolescent Type 1 Diabetes Cardio‐Renal Intervention Trial, the determination of UACR has been suggested to exert more prognostic value for cardiovascular complications of diabetes.^35^ Here, our result revealed the UACR as an independent and contributing factor for the reduced GLS, showing a significant inverse relationship with GLS. These data were consistent with Mochizuki et al's and Levelt et al‘s research.^32^, ^36^ To some extent, the theory of “common soil” could partly interpret those findings, and it is thus more logical that microvascular complications, especially albuminuria, may serve as an early clinical warning of vascular damage and endothelial dysfunction, illustrating an increased heart burden and impairing cardiac performance.

### 4.4 Serum lipids and LV longitudinal myocardial function

More recent evidence suggests that under hyperglycemia conditions, the increased fatty acid uptake by myocardium exceeds the oxidation capacity of nonadipose tissues to free fatty acids, which results in the excessive deposition within heart in the form of triglyceride known as lipotoxicity.^37^ In terms of molecular mechanism, the transition of cardiac metabolism leading to lipid toxic injury greatly contributes to the pathogenesis of diabetic cardiomyopathy. Generally, triglyceride is relatively inert and thus does not directly mediate lipotoxicity but rather the intermediate product of triglyceride primarily responsible for cardiac dysfunction.^38^ However, there exist debates on the role of hypertriglyceridemia as an independent cardiovascular risk factor.^39^ More recent epidemiological studies tended to support the cascading effect of triglyceride that the excessive production of proinflammatory cytokines and the reduced endothelial nitric oxide biosynthesis jointly contribute to cardiac microcirculation dysfunction.^40^ Depending on the results of magnetic resonance spectroscopy, research conducted by Ng et al confirmed an association of high myocardial triglyceride content with LV myocardial longitudinal strain.^41^ In a study of type1 diabetes by Vinereanu et al, LDL rather than HbA1c was found the only independent predictor of the cutoff value of 18.6% of abnormal LV GLS,^42^ which was consistently revealed by other authors in T2DM, followed by the inverse relation of LV longitudinal function to LDL.^43^ In contrast, another study covering 144 type 1 and type 2 diabetes has identified that hypertriglyceridemia but not low‐ and high‐density lipoprotein, resulted in further damage to GLS.^36^ Similarly, a study carried out on a population of Chinese ethnicity in Taiwan has emphasized that triglycerides potentially played an adverse effect on the progress of cardiovascular, free from other lipid parameters.^44^ Similar outcomes were also obtained in Chinese patients with T2DM and proved in the present study of ours, illustrating a possible involvement of triglyceride in the occurrence and development of LV systolic function impairment. Additionally, BMI was not an independent risk factor as identified here, which showed bias compared to the study of Tseng et al, where BMI was determined as a significant predictor for heart disease in T2DM patients in Taiwan.^45^ This discrepancy could perhaps be explained by the selection bias, lack of adequate sample size, and the deficiency in accuracy of BMI measurements. Notably, the patients with reduced GLS were more prone to receiving lipid‐lowering drugs (especially statins) compared to those with normal GLS. As was reported, statins contributed a lot to the correlation between glycemic control, dyslipidemia, and cardiovascular disease.^46^ Another nonnegligible observation was the increased exogenous insulin use but the decreased metformin use on subjects with reduced GLS, which has exhibited protective effects on the development of hypertension^47^ and heart failure.^48^ Despite no statistical effect of medication on GLS was observed, the results should be interpreted as the important confounding effects of the use of statins and antidiabetic drugs.

Expectedly, the present study has certain clinical implications. It demonstrated that glucotoxicity and lipotoxicity promoted by metabolic disturbances were the triggers to the hallmark pathological transformations in the diabetic heart. In addition, microvascular complications may provide incremental diagnostic benefit in identifying transformations in subclinical myocardial function, which in turn allows for an earlier identification of risk factors to prevent heart failure progression. However, the data on the correlation between subclinical LV dysfunction and microvascular complications in diabetic patients are currently still limited. The findings in this study further display a close relationship between microvascular complications and the reduced GLS. In addition, not only the reduced GLS concerning the uncontrolled glucose and serum lipid levels was further confirmed, but the newly clinical significance of HbA1c was also a focus. The results of the ROC curve indicated HbA1c >8.8% provided a reasonable specificity to identify high‐risk patients with diabetes. Conclusively, our research demonstrates the advantage of wide clinical applicability that evaluation of HbA1c, including blood lipids, is a convenient and quick screening tool with low cost. It not only could identify altered metabolic homeostasis in earlier individuals but could also serve as an initial warning for clinical monitoring of myocardial dysfunction, emphasizing the intensive control of glucose and blood lipids as a potentially promising strategy in patients with type 2 diabetes without the history of cardiovascular events.

The limitations of this study should also be considered. First, as a cross‐sectional study, whether strict glycemic control is associated with improvement in GLS remains unclear, and further observation by follow‐up is required. Second, invasive coronary angiography was not performed on patients without coronary artery disease. In addition, this retrospective study was carried out for hospitalized type 2 diabetes patients, with a single‐center population enrolled, so that selection bias cannot be fully ruled out, resulting in the limited extensibility of the research results. Finally, considering the epidemic prevention and the control measures that may affectt the lifestyle and therapeutic drugs of patients, stratified cluster sampling is recommended so that large samples will be adopted for prospective research to reduce bias in future studies.

In conclusion, the present study illustrated a robust correlation between diabetic complications with subclinical LV dysfunction. In addition, HbA1c and hypertriglyceridemia could serve as independent risk factors for the early stage of LV myocardial dysfunction in asymptomatic T2DM patients. More important, HbA1c may exert prognostic significance for the progression of myocardial damage.

## CONFLICT OF INTEREST

No potential conflict of interest relevant to this article was reported.
